# The burden of persistent symptoms after COVID-19 (long COVID): a meta-analysis of controlled studies in children and adults

**DOI:** 10.1186/s12985-024-02284-3

**Published:** 2024-01-11

**Authors:** Ahmed Azzam, Heba Khaled, Neveen Refaey, Shorouk Mohsen, Ola Ali El-Emam, Nada Dawood, Hebatalla A. Ahmed, Omar A. Soliman, Sana Mostafa, Heba Ramadan, Maha Mosa, Amora Omar Ibrahim Elmowafy, Shimaa Mohamed Abdou Rizk, Ahmed Zaki, Mostafa Hussien, Ameer Ahmed, Ahmad Ashraf Ezzat, Fatma E. Hassan

**Affiliations:** 1 Department of Microbiology and Immunology, Faculty of Pharmacy, Helwan University, Cairo, Egypt; 2 Department of Biochemistry, Faculty of Pharmacy, Cairo University, Cairo, Egypt; 3 Department of Physical Therapy for Women’s Health, Faculty of Physical Therapy, Cairo University, Cairo, Egypt; 4 Public Health and Preventive Medicine Department, Faculty of Medicine, Mansoura University, Mansoura, Egypt; 5 Clinical Pathology Department, Faculty of Medicine, Mansoura University, Mansoura, Egypt; 6 Community Medicine Department, Faculty of Medicine, Suez Canal University, Ismailia, Egypt; 7 Department of Public Health and Community Medicine, Faculty of Medicine, Kafr-Elsheikh University, Kafr-Elsheikh, Egypt; 8 Department of Clinical Pharmacy, Alexandria, University Main Teaching Hospital, Alexandria, Egypt; 9 Human Genetics Department, Medical Research Institute, Alexandria University, Alexandria, Egypt; 10 Oral Biology Department, Faculty of Dentistry, Cairo University, Cairo, Egypt; 11 Pharmacy Department, Agamy Medical District, Ministry of Health and Population, Agamy, Alexandria Egypt; 12 Otolaryngologist, Qeft Teaching Hospital, Qena, Egypt; 13 Medical Surgical Nursing Department, Faculty of Nursing, Mansoura University, Mansoura, Egypt; 14 Faculty of Medicine, Cairo University, Cairo, Egypt; 15 Faculty of Medicine, Suez Canal University, Suez City, Ismailia Governorate Egypt; 16 Faculty of Medicine, Minia University, Minya, Egypt; 17 Faculty of Pharmacy, Minia University, Minya, Egypt; 18 Medical Physiology Department, Kasr Alainy, Faculty of Medicine, Cairo University, Giza, 11562 Egypt; 19 Department of Physiology, General Medicine Practice Program, Batterjee Medical College, 21442 Jeddah, Saudi Arabia

## Abstract

**Background:**

Previous meta-analyses estimating the prevalence of the post-COVID-19 condition (PCC) were confounded by the lack of negative control groups. This may result in an overestimation of the prevalence of those experiencing PCC, as these symptoms are non-specific and common in the general population. In this study, we aimed to compare the burden of persistent symptoms among COVID-19 survivors relative to COVID-19-negative controls.

**Methods:**

A systematic literature search was conducted using the following databases (PubMed, Web of Science, and Scopus) until July 2023 for comparative studies that examined the prevalence of persistent symptoms in COVID-19 survivors. Given that many of the symptoms among COVID-19 survivors overlap with post-hospitalization syndrome and post-intensive care syndrome, we included studies that compare the prevalence of persistent symptoms in hospitalized COVID-19 patients relative to non-COVID-19 hospitalized patients and in non-hospitalized COVID-19 patients relative to healthy controls that reported outcomes after at least 3 months since infection. The results of the meta-analysis were reported as odds ratios with a 95% confidence interval based on the random effects model.

**Results:**

Twenty articles were included in this study. Our analysis of symptomatology in non-hospitalized COVID-19 patients compared to negative controls revealed that the majority of symptoms examined were not related to COVID-19 infection and appeared equally prevalent in both cohorts. However, non-COVID-19 hospitalized patients had higher odds of occurrence of certain symptoms like anosmia, ageusia, fatigue, dyspnea, and brain fog (*P* < 0.05). Particularly, anosmia and ageusia showed substantially elevated odds relative to the negative control group at 11.27 and 9.76, respectively, *P* < 0.05. In contrast, analysis of hospitalized COVID-19 patients compared to those hospitalized for other indications did not demonstrate significantly higher odds for the tested symptoms.

**Conclusions:**

The persistent symptoms in COVID-19 survivors may result from hospitalization for causes unrelated to COVID-19 and are commonly reported among the general population. Although certain symptoms exhibited higher odds in non-hospitalized COVID-19 patients relative to controls, these symptoms are common post-viral illnesses. Therefore, the persistent symptoms after COVID-19 may not be unique to SARS-CoV-2. Future studies including well-matched control groups when investigating persistent symptoms in COVID-19 survivors are warranted to draw a firm conclusion.

**Supplementary Information:**

The online version contains supplementary material available at 10.1186/s12985-024-02284-3.

## Introduction

Since the initial outbreak of the severe acute respiratory syndrome coronavirus 2 (SARS-CoV-2) pandemic, there has been a growing trend in the potential long-term implications of Coronavirus disease 2019 (COVID-19), often known as long COVID or post-COVID-19 condition (PCC) [1]. Per the World Health Organization (WHO), the majority of patients who get COVID-19 completely recover. However, contemporary data indicates that about 10–20% of COVID-19-infected patients experience symptoms that can be designated as PCC [2]. Over 200 distinctive signs and symptoms were identified as relevant to PCC [3].

Even though the COVID-19 pandemic has been ongoing for nearly 4 years, there is still no consensus among various recommendations as well as organizations on an accurate depiction of PCC [4–7]. The WHO’s definition of PCC was developed through a Delphi process, in which PCC comes up in individuals with a history of most likely or verified SARS-CoV-2 infection, usually 3 months upon onset, with symptoms lasting at least 2 months and being unlikely to be attributed to another clinical condition [8]. However, this definition may be skewed because it does not take illness severity [9] into account and is contingent on a history of potential SARS-CoV-2, which is not objective. Furthermore, the elements addressing the timing and duration of symptoms do not meet consensus requirements. Because of this, continual discussion and the incorporation of contemporary proof are required to advance this definition [8].

The previous meta-analyses that aimed to estimate PCC prevalence were confounded by the lack of negative control groups [10–13]. This results in an overestimation of the prevalence of those experiencing PCC, with preliminary estimates spanning from 45 to 80% [12, 13]. Without negative control groups, it is challenging to properly compare the burden of long COVID and the symptoms profile among individuals who test positive and those who test negative for SARS-CoV-2 because some of the reported long COVID symptoms are non-specific and pervasive in the population [14]. Moreover, these individuals might exhibit the symptoms as a result of pre-existing health disorders or the wider implications of the pandemic, thereby making it more difficult for researchers to draw accurate conclusions without the inclusion of control groups [14].

On top of that, the recent scientific literature points out that hospitalization could potentially play a crucial role in the development of PCC. The following evidence backs up this claim: First, previous meta-analyses indicated that hospitalized COVID-19 patients had a greater prevalence of PCC than non-hospitalized patients [11, 13, 15]. Second, the risk of developing PCC is significantly higher in hospitalized patients, with a 2.48 odds ratio (OR) compared to non-hospitalized patients [16]. Furthermore, comorbidities and pre-existing medical conditions such as anxiety, depression, asthma, chronic obstructive pulmonary disease (COPD), immunosuppression, and ischemic heart disease had a higher odds ratio of acquiring PCC compared to non-comorbid COVID-19 patients [16].

To circumvent the limitations of past studies and appropriately address the burden of persistent symptoms after COVID-19 relative negative control PCC, we carried out this systematic review and meta-analysis of research studies that involved COVID-19 positive individuals in comparison to COVID-19 negative individuals. Given the potential effect of hospitalization on the prevalence of symptoms after COVID-19, we included studies that compare the prevalence of persistent symptoms following COVID-19 in hospitalized COVID-positive patients relative to those who were hospitalized for reasons other than COVID-19 infection and in non-hospitalized COVID-positive individuals relative to healthy controls. Further, to find out the impact of comorbidities, we did a subgroup analysis of the studies that were comorbidity-matched.

## Methods

### Search strategy

A comprehensive literature search was conducted in the following databases: PubMed, Scopus, and Web of Science, concerning the relevant studies until July 2023. The Preferred Reporting Items for Systematic Reviewers and Meta-analysis (PRISMA) criteria were followed. The reference lists of relevant articles were reviewed for additional studies. The checklist of items to include when reporting a systematic review or meta-analysis and the complete search strategy were presented in Additional file 1: Tables S1 and S2, respectively; see the additional file.

### Study selection

#### Studies were included if they met the following criteria

(a) Articles written in English. (b) Peer-reviewed comparative studies that compare the prevalence of persistent symptoms in COVID-positive and COVID-negative individuals after at least 3 months post-COVID-19 (as defined by WHO). (c) Patients who were hospitalized for COVID-19 compared to patients who were hospitalized due to other infections or causes or (d) non-hospitalized individuals versus healthy controls. (e) Laboratory-confirmed COVID-19; and (f) reporting of follow-up as mean, median, or a set interval after symptom onset, diagnosis, acute illness, or initial computed tomography (CT) chest imaging.

### Exclusion criteria for this meta-analysis

The subsequent studies were excluded from the analysis: (a) Studies that reported the prevalence of persistent symptoms in patients hospitalized with COVID-19 versus healthy controls. (b) Studies where the duration of follow-up could not be determined. (c) Studies in which all patients were not assessed for a minimum of 12 weeks. (d) Studies that specifically recruited sub-groups of patients, such as those with diabetes or autoimmune conditions.

Three independent reviewers selected eligible articles based on the aforementioned eligibility criteria. Discrepancies were resolved through consultation with another author.

### Data extraction

Three independent reviewers extracted data from the included studies using a standardized data extraction form. Any discrepancies were settled by discussion.

From each included study, the following were extracted: (a) Study details including study design, study aim, study population, country of origin, number of cases (COVID-19 survivors), number of controls (COVID-19 negative individuals), duration, and method of follow-up. (b) Participants’ characteristics, such as age, sex, presence of comorbidities, and hospitalization status. (c) Outcomes associated with post COVID-19 condition in both the case and control groups.

### Quality assessment

The assessment of the studies’ quality was carried out using a modified scale based on the Newcastle–Ottawa Scale of case–control studies displayed in Additional file 1: Table S3. The items on the modified scale were: (a) Did the sample size exceed 10,000 or not? (b) How was the ascertainment of COVID-19 infection made; via polymerase chain reaction (PCR) test, serology test, both, or none? c) Was the same method of ascertainment used for cases and controls or not? (d) What was the definition of control; no history of disease or not described? (e) Was the non-response rate the same for both groups? (f) Did the study control for comorbidity, age, and gender or not? g) If control for comorbidity, age, and gender was not achieved, was the study adjusted for confounding factors using multivariate analysis or stratification?

Two independent reviewers assessed the quality of the studies, and discrepancies were resolved through consultation with a third author.

### Data synthesis

Results were reported as odds ratios with a 95% confidence interval (CI) based on the random effects model. We used the I^2^ statistic to measure statistical heterogeneity between the results of the studies, with values less than 50% indicating low heterogeneity and values greater than or equal to 50% indicating high heterogeneity. Since different studies reported varying symptom patterns and numbers, meta-analysis was only performed for symptoms with data from at least three studies. Subgroup analyses were conducted based on age categories and the studies that were matched in terms of comorbidities. In addition, sensitivity analyses were conducted using a one-leave-out approach to identify any outlier studies that may have significantly affected the overall results. Testing for publication bias by funnel plots and Egger's tests was considered for analysis in more than 10 studies [17]. All statistical analysis was performed using Comprehensive Meta-Analysis version 3.0 (Biostat, Englewood, NJ, USA).

## Results

### Study selection and characteristics of included studies

**Fig. 1:**
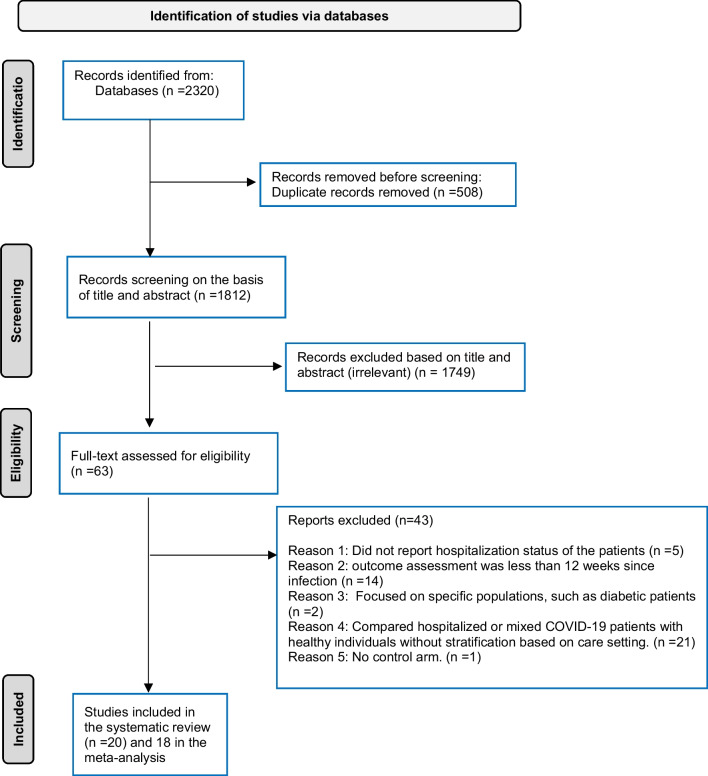
PRISMA flow diagram depicting the selection of publications

In total, 2320 titles were identified through database searches, of which 20 were included in this review [16–35] (Fig. 1). The list of excluded studies with reasons for exclusion was presented in Additional file 1: Table S4. As displayed in Table 1, the current systematic review comprised 20 studies representing a variety of countries. Each of the subsequent countries had one research conducted: Sweden [36], Switzerland [29], Germany [18], Italy [19], Slovakia [25], Norwegian [9], France [33], Netherlands [34], Denmark [27], and Spain [30]. Two studies were done in Norway [22, 31], while another 2 studies were reported in Israeli [21, 26], four studies were performed in the UK [23, 24, 28, 32] and one study in the USA [20]. One study involved participants from many nations including Argentina, Canada, Costa Rica, Italy, Paraguay, Singapore, Spain, and the United States [35].

**Table 1:** Characteristics of included studies

Author	Country	Age category	Age (Years) Mean ± SD/Median (IQR)	Females%	Study design	Cases (No.)	Comorbid (%)	Control (No.)	Follow-up since diagnosis (Median)	Assessment method
*A: Non-hospitalized COVID-19 versus negative control*										
Larsson [36]	Sweden	Adults	45 ± 12	86.4	RC (Matched age)	1584	Non-significant except for mental disorder (higher in the negative arm)	7185	> 12 weeks	Self-reported questionnaire
Radtke [29]	Switzerland	Children	11 (9–13)	54	PC	109	–	1226	6 months	Both of Self-reported questionnaire and Physician assessment
Blankenburg [18]	Germany	Adolescents and young adults	15 (14–17)	55	PC (Matched age and gender)	188	–	1365	10 months	Self-reported questionnaire
Boscolo-Rizzo [19]	Italy	Adults	49 (21–70)	59	PC (Matched age and gender)	61	Matched	61	1 year	Both of Self-reported questionnaire and Physician assessment
Fjelltveit [22]	Norway	Mixed	44 (0–81)	53	PC (Matched age, gender and comorbidity)	220	Matched	182	12 months	Both of Self-reported questionnaire and Physician assessment
Pereira [28]	UK	Children	11–17	62.3	PC (Study invitation, by age, gender, region, and date of testing)	6407	–	6542	6 months	Self-reported questionnaire
Mizrahi [26]	Israel	Mixed	25 (12–43)	50.6	RC (Matched age, gender and comorbidity)	170,280	Matched	170,280	6–12 months	Physician assessment
Gorecka [23]	UK	Adults	45 ± 13	53	PC (Matched age and gender)	19	Both groups are healthy	10	> 12 weeks	Clinical examination (Imaging)
Joy [24]	UK	Adults	37 (18–63)	58	PC (Matched Age, gender, and ethnicity)	74	Non-significant except for asthma/COPD which is higher in the case than control	75	6 months	Clinical examination (Imaging)
Liptaka [25]	Slovakia	Adults	40 (32–52)	70	PC (Unmatched)	109	–	132	7 months	Self-reported questionnaire
Selvakumar [9]	Norwegian	Adolescents and Young adults	12–25	60.2	PC (Unmatched)	382	Any comorbidity is 37% for COVID-19-negative compared to 24% for COVID-19 positive	85	6 months	Both of Self-reported questionnaire and Physician assessment
Soraas [31]	Norway	Adults	48.5 ± 13.5	56.8	PC (Unmatched)	676	–	6006	3–8 months	Self-reported questionnaire
Stephenson [32]	UK	Children	11–17	63.5	PC (Month of test, age, gender, and geographical region)	3065	–	3739	3 months	Self-reported questionnaire
Funk (a) [35]	Argentina, Canada, Costa Rica, Italy, Paraguay, Singapore, Spain, and the United States	Children	3 (0–10)	45	PC (Matched hospitalization status, country, and recruitment date)	1295	Matched	1321	3 months	Self-reported questionnaire
Tarazona [33]	France	Adults	45.8 ± 14.9	59.4	PC (Matched age, gender and comorbidity)	96	Non-significant except for cardiological conditions (higher in case)	81	12 months	Self-reported questionnaire
Van der Maaden [34]	Netherlands	Adults	49 (37–61)	63.7	PC (Matched age, gender, and comorbidity)	6,614	Matched	1330	3 months	Both of Self-reported questionnaire and Physician assessment
*B: Hospitalized COVID-19 versus non COVID hospitalized control*										
Funk (b) [35]	Argentina, Canada, Costa Rica, Italy, Paraguay, Singapore, Spain, and the United States)	Children	4 (0–12)	45	PC (Matched hospitalization status, country, and recruitment date)	391	Matched	380	3 months	Self-report questionnaire
Elkan [21]	Israel	Adults	58.5 (49.8–68.3)	56.1	RC (Matched age and gender)	42	Matched in all except for chronic lung disease (higher in non-COVID control)	42	9 months	Self-report questionnaire
Castro [20]	USA	Adults	63 (50–76)	47	RC (Unmatched)	6619	–	36,342	3–5 months	Physician assessment
Nersesjan [27]	Denmark	Adults	56.8 ± 14	42	PC (Matched age, gender, and ICU status from the same hospital, including patients in the ICU)	85	Matched	61	6 months	Both of Self-reported questionnaire and Physician assessment
Rivera-Izquierdo [30]	Spain	Adults	61.2 ± 14.3	42.6	PC (Matched institution and date of admission)	453	Matched	453	12 months	Self-report questionnaire

*No.* number, *RC* retrospective cohort, *PC* prospective cohort, *Vs.* versus, *ICU* intensive care unit, *SD* standard deviation, *IQR* interquartile range

### Stratification of the included studies based on the hospitalization status of COVID-19 patients and negative control

The twenty included studies resulted in 21 estimates, with Funk et al. aiming to estimate PCC for non-hospitalized children relative to the negative control and also for hospitalized children relative to non-COVID-19 hospitalized children [35].

Sixteen of the twenty-one studies assessed PCC in non-hospitalized COVID-19 patients in comparison to negative controls [9, 18, 19, 22–26, 28, 29, 31–36]. The remaining five studies addressed PCC in COVID-19 patients who were hospitalized [20, 21, 27, 30, 35]. Three studies compared hospitalized COVID-19 patients to those hospitalized for other indications without specifying the cause of hospitalization [20, 30, 35]. Another study evaluated hospitalized COVID-19 patients in comparison to non-COVID-19 hospitalized patients due to pneumonia [21]. One study investigated hospitalized COVID-19 patients relative to those hospitalized with acute myocardial infarction [27].

### Age category

When categorizing studies by age, four studies were performed on children [28, 29, 32, 35], two studies involved adolescents and young adults [9, 18], 12 studies included adults [19–21, 23–25, 27, 30, 31, 33, 34, 36] and only 2 studies included a wide age range [22, 26].

### Follow-up since COVID-19 onset

The included studies in this research encompassed a diverse range of follow-up durations aimed at monitoring participants’ conditions after diagnosis. Some studies allowed for extended, short-term observation [23, 36]. Most of the studies focused on a 6-month follow-up, providing insights into the mid-term progression of the studied conditions [9, 24, 27–29]. However, other studies conducted comprehensive 7-month, 9-month, and 10-month follow-up periods, [18, 21, 25]. Effectively, more studies focused on the long-term progression of the condition [22, 30, 33]. Some studies implemented a flexible approach toward follow-up and had a follow-up period of 6–12 months, 3–8 months, and 3–5 months [20, 26, 31]. It’s worth noting that some studies had the shortest median follow-up period of 3 months [32, 34, 35].

### Quality assessment of the included studies

The detailed score for each included study was presented in Additional file 1: Table S5. Fifteen of the twenty studies have a score of more than six out of 10 and are considered of fair quality. While five studies had a score of five or less and may have a high risk of bias either because of a small sample size, different rates of non-response, subjective assessment of outcomes, or non-adjustment for confounding factors [18, 19, 21, 29, 33].

### Post-COVID consequences in non-hospitalized COVID-19 patients compared to negative controls

**Fig. 2:**
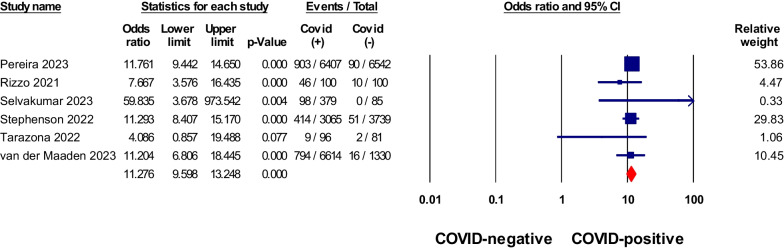
Meta-analysis of odds ratios of anosmia in non-hospitalized COVID-19 relative to negative control

**Fig. 3:**
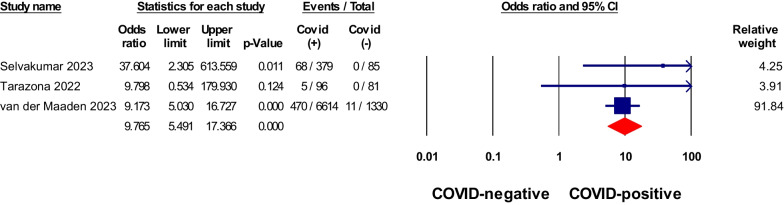
Meta-analysis of odds ratios of ageusia in non-hospitalized COVID-19 relative to negative control

**Fig. 4:**
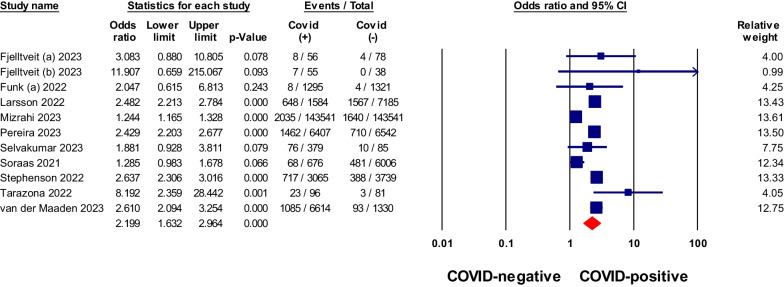
Meta-analysis of odds ratios of dyspnea in non-hospitalized COVID-19 relative to negative control

**Fig. 5:**
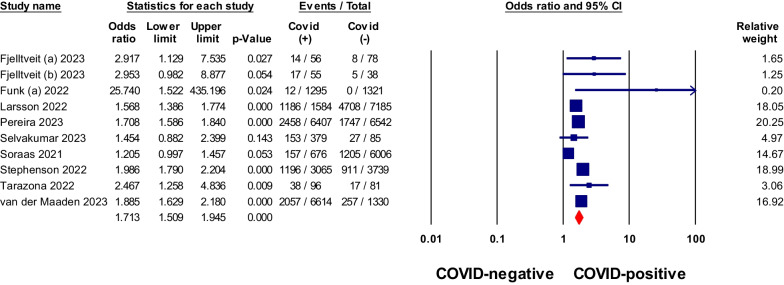
Meta-analysis of odds ratios of fatigue in non-hospitalized COVID-19 relative to negative control

**Fig. 6:**
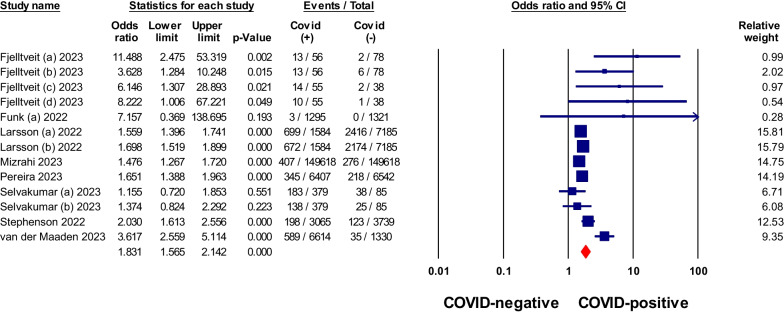
Meta-analysis of odds ratios of brain fog or memory deficits in non-hospitalized COVID-19 relative to negative control

Non-hospitalized patients with COVID-19 were found to have a stronger association with certain symptoms compared to negative controls (Table 2). These symptoms included anosmia, ageusia, dyspnea, fatigue, and brain fog or confusion, with pooled odds ratios greater than 1, as shown in Figs. 2, 3, 4, 5 and 6. Anosmia had the highest odds ratio of 11.27, followed by ageusia with 9.76. Some manifestations, such as chest pain, dizziness, skin conditions, myalgia/arthralgia, and ear problems, showed slightly elevated odds ratios of 1.9, 1.37, 1.42, 1.25, and 1.35, respectively, *P* < 0.05. After matching for comorbidities, the pooled odds ratios for certain symptoms changed. Anosmia, dyspnea, fatigue, and brain fog continued to exhibit significantly higher odds ratios in COVID-19 patients even after matching for comorbidities (4.91, 2.29, 2.2, and 2.91, *P* < 0.05), respectively. However, chest pain, skin conditions, myalgia/arthralgia, and ear problems no longer showed a significant association.

**Table 2:** Meta-analysis of odds ratios for signs, symptoms, and conditions in non-hospitalized COVID-19 patients compared to negative controls, with subgroup analysis by comorbidity matching

Sign/symptom/condition	Overall			Matched comorbidity		
	Studies No	Pooled odds ratio (95% CI)	I^2^%	Studies No	Pooled odds ratio (95% CI)	I^2^%
Abdominal pain	7	0.96 (0.76–1.23)	87.4	–	–	–
Diarrhea	5	1.14 (0.9–1.42)	63.1	–	–	–
Nausea/Vomiting	5	0.95 (0.81–1.12)	51.7	3	0.89 (0.8–0.99)	9.8
Heartburn/Stomachache	3	1.02 (0.9–1.16)	0	–	–	–
Sore throat	9	0.85 (0.67–1.08)	90.5	4	1.04 (0.95–1.14)	0
Fatigue	11	1.7 (1.51–1.93)	68.5	5	2.2 (1.6–3.03)	21.7
Headache	11	1.13 (0.89–1.42)	95.3	4	1 (0.83–1.21)	60.9
Fever	7	1.02 (0.82–1.26)	35.8	–	–	–
Dizziness	6	1.37 (1.08–1.75)	90.03	3	1.13 (1.05–1.2)	0
Anosmia	6	11.27 (9.59–13.24)	0	3	4.91 (1.48–16.3)	0
Ageusia	3	9.76 (5.49–17.36)	0	–	–	
Congested or runny Nose	4	0.89 (0.78–1.02)	0	3	0.89 (0.78–1.02)	0
Cough	10	0.95 (0.8–1.12)	78.3	4	0.98 (0.94–1.03)	0
Dyspnea	11	2.19 (1.63–2.96)	95.9	6	2.52 (1.41–4.52)	90.4
Anxiety	3	1.09 (0.98–1.21)	0	–	–	–
Sleep disorders	6	1.08 (0.95–1.22)	23.1	–	–	–
Depression	6	0.91 (0.72–1.16)	67.3	–	–	
Brain fog	18	1.85 (1.58–2.16)	79	10	2.91 (2.05–4.15)	80.3
Tachycardia/palpitation	4	1.46 (1.06–2.03)	90.8	–	–	–
Chest pain	8	1.9 (1.28–2.82)	97.3	4	1.74 (0.85–3.56)	87.2
Myalgia/Arthralgia	12	1.25 (1.07–1.45)	90.8	4	1.1 (0.97–1.23)	75.6
Skin conditions	4	1.42 (0.85–2.4)	74.9	–	–	–
Ear problems	5	1.35 (1.24–1.46)	0	3	1.06 (0.74–1.51)	0

*No.* number, *CI* Confidence interval

### Post-COVID consequences in non-hospitalized children with COVID-19 compared to negative controls

Anosmia, fatigue, brain fog or confusion, and dizziness were found to be associated with non-hospitalized children with COVID-19 compared to negative controls (*P* < 0.05) as shown in Table 3. Especially anosmia had a very high odds ratio of 10.45. However, the pooled odds ratios for specific symptoms varied when comorbidities were matched. Brain fog and dizziness were no longer significant (*P* > 0.05), while there were not enough studies on fatigue to be tested.

**Table 3:** Meta-analysis of odds ratios for signs, symptoms, and conditions in non-hospitalized children with COVID-19 compared to negative controls, with comorbidity-matched subgroup analysis

Sign/Symptom/Condition	Overall			Matched comorbidity		
	Studies No	Pooled odds ratio (95% CI)	I^2^%	Studies No	Pooled odds ratio (95% CI)	I^2^%
Abdominal pain	4	0.99 (0.74–1.33)	93.1	–	–	–
Sore throat	5	1.22 (1.01–1.48)	67.6	3	1.30 (0.84–2.02)	72.5
Fatigue	4	1.84 (1.56–2.16)	66	–	–	–
Headache	6	1.24 (0.89–1.73)	94.5	3	0.88 (0.78–0.98)	0
Fever	3	0.96 (0.76–1.21)	0	–	–	–
Dizziness	5	1.41 (1.08–1.85)	81.5	3	0.97 (0.76–1.22)	0.46
Anosmia	3	10.45 (7.46–14.62)	0	–	–	–
Congested or runny nose	4	0.68 (0.40–1.16)	0	3	0.65 (0.36–1.17)	0
Cough	6	0.97 (0.88–1.08)	0	3	0.91 (0.83–1.00)	0
Dyspnea	5	1.56 (0.97–2.51)	96.7	3	0.90 (0.75–1.08)	8.7
Anxiety	3	0.80 (0.64–1.01)	0	–	–	–
Brain fog	5	1.63 (1.24–2.15)	51	3	1.10 (0.54–2.23)	0.1
Chest pain	4	1.25 (0.70–2.21)	92.8	3	0.88 (0.76–1.03)	0
Myalgia/arthralgia	7	1.21 (0.75–1.95)	92.6	5	0.83 (0.61–1.12)	13.9

*No.* number, *CI* confidence interval

### Post-COVID consequences in non-hospitalized adults with COVID-19 compared to negative controls

Abdominal pain was significantly greater in the negative control group (OR 0.83) (*P* < 0.05). In contrast, symptoms significantly associated with non-hospitalized COVID-19 compared to controls included fatigue, dyspnea, brain fog, anosmia, chest pain, sleep disturbances, and tachycardia (OR 1.68, 1.99, 2.29, 4.95, 1.44, 1.14, and 1.34 *P* < 0.05, respectively). However, subgroup analyses matching for comorbidities revealed that associations with sleep disturbances, chest pain, tachycardia, and abdominal pain were no longer statistically associated in non-hospitalized COVID-19 patients, as presented in Table 4**.**

**Table 4:** Meta-analysis of odds ratios for signs, symptoms, and conditions in non-hospitalized adults with COVID-19 compared to negative controls, with co-morbidity-matched subgroup analysis

Sign/symptom/condition	Overall			Matched comorbidity		
	Studies No	Pooled odds ratio (95% CI)	I^2^%	Studies No	Pooled odds ratio (95% CI)	I^2^%
Abdominal pain	5	0.83 (0.69–0.99)	83	3	0.94 (0.85–1.04)	62.9
Nausea or vomiting	6	0.95 (0.84–1.07)	1.08	4	0.91 (0.82–1.00)	0
Sore throat	6	0.79 (0.57–1.09)	91.8	4	0.98 (0.88–1.09)	0
Diarrhea	3	1.04 (0.43–2.51)	70.1	–	–	–
Fatigue	6	1.68 (1.36–2.07)	76	4	1.94 (1.69–2.23)	0
Headache	6	0.91 (0.76–1.09)	90.2	4	1.00 (0.86–1.16)	78.3
Fever	3	0.99 (0.63–1.58)	72.8	–	–	–
Dizziness	3	1.18 (1.04–1.35)	52.4	–	–	
Anosmia	5	4.95 (2.64–9.31)	79.4	5	4.95 (2.64–9.31)	79.4
Sleep disorders	4	1.14 (1.04–1.26)	0	3	1.16 (0.96–1.39)	0
Congested or runny nose	3	0.90 (0.79–1.04)	0	–	–	–
Cough	6	0.91 (0.75–1.11)	88.7	4	1.01 (0.94–1.07)	3.3
Dyspnea	8	1.99 (1.52–2.61)	90.5	6	2.05 (1.49–2.81)	85.4
Depression	4	1.03 (0.90–1.19)	30.3	3	0.92 (0.77–1.10)	0
Anxiety	4	1.01 (0.88–1.16)	40.6	3	0.94 (0.79–1.12)	19.6
Brain fog/confusion/difficulty in concentration	12	2.29 (1.88–2.78)	90.1	10	2.63 (2.09–3.30)	81.1
Chest pain	5	1.44 (1.02–2.03)	95.1	4	1.21 (0.94–1.56)	87
Tachycardia	4	1.34 (1.04–1.73)	87.9	3	1.21 (0.98–1.5)	70
Myalgia/arthralgia	8	1.11 (0.98–1.25)	85.4	5	1.10 (0.97–1.25)	73.6

*No.* number, *CI* confidence interval

### Post-COVID consequences in hospitalized COVID-19 patients compared to non-COVID-19 hospitalized patients

Of the five symptoms tested, only headache and sleep disorders (OR 0.86 and 0.89, respectively, *P* < 0.05) showed significantly lower odds of occurrence in hospitalized COVID-19 patients compared to patients hospitalized for other reasons, as shown in Table 5. The other three symptoms, brain fog, anxiety, and fatigue, did not have significantly higher odds in COVID-19 patients, although brain fog and anxiety had slightly elevated odds ratios of 1.19 and 1.04, respectively. In contrast, the odds ratios for fatigue were slightly lower at 0.94.

**Table 5:** Meta-Analysis of odds ratios of post-COVID consequences in hospitalized COVID-19 patients versus non-COVID-19 hospitalized patients

Sign/symptom/condition	Hospitalized COVID-19 versus hospitalized for other indications		
	Studies (No.)	Pooled odds ratio (95% CI)	I^2^%
Fatigue	4	0.94 (0.65–1.36)	57.2
Brain fog	3	1.19 (0.51–2.76)	62
Headache	3	0.86 (0.77–0.96)	0
Anxiety	4	1.04 (0.51–2.10)	78.2
Sleep disorders	3	0.89 (0.8–0.98)	0

*Vs.* versus, *No.* number, *CI* confidence interval

### Included studies comparing myocardial parameters (structure, function, tissue characteristics, and perfusion) between patients with mild COVID-19 and COVID-negative healthy controls

As shown in Table 6, two studies aimed to assess myocardial structure, function, and tissue characterization in those with mild COVID-19 syndrome and COVID-negative healthy controls after more than 12 weeks and 6 months since infection.

**Table 6:** Included studies comparing cardiac parameters (Structure, function, tissue characterization, and perfusion) between patients with mild COVID-19 syndrome and COVID-negative healthy controls

Author	Time since diagnosis	Case/Control number	Cardiac parameters tested	Study findings
Gorecka [23]	> 12 weeks	20/10	Myocardial energetics: phosphocreatine to ATP ratio Cardiac structure: biventricular volumes Cardiac function: biventricular ejection fractions and global longitudinal strain Tissue characterization: T1 mapping and late gadolinium enhancement Perfusion: myocardial rest and stress blood flow, and myocardial perfusion reserve	No significant difference
Joy [24]	6 months	74/75	Cardiac structure: left ventricular volumes, mass, and atrial area Cardiac function: ejection fraction, global longitudinal shortening, and aortic dispensability Tissue characterization: T1, T2, extracellular volume fraction mapping, and late gadolinium enhancement Biomarkers: troponin, and N-terminal pro–B-type natriuretic peptide	No significant difference

Both studies assessed age-, sex-, and comorbidity-matched participants

Gorecka et al. [23] found that most patients with a long COVID-19 syndrome and no previous cardiovascular disease did not show any signs of abnormalities in their myocardial energetics, structure, function, blood flow, or tissue characteristics. Likewise, Joy et al. [24] demonstrated that there was no significant difference in cardiovascular abnormalities between seropositive individuals and those who were seronegative, even among otherwise healthy participants.

### Post-COVID consequences in COVID-positive patients relative to COVID-negative controls across all included studies overall and stratified by patients’ age category

Overall, compared to negative control, anosmia, dyspnea, chest pain, brain fog, dizziness, tachycardia/palpitation, ageusia, fatigue, and myalgia/arthralgia were significantly higher in COVID-positive patients (pooled OR 6.9, 2.1, 1.72, 1.77, 1.35, 1.34, 6.57, 1.53, and 1.22, respectively). Nevertheless, when age-based subgroup analysis was done, there was no statistically significant difference in chest pain in both the adult and children’s categories (Additional file 1: Table S6).

### Sensitivity analysis and publication bias testing

**Fig. 7:**
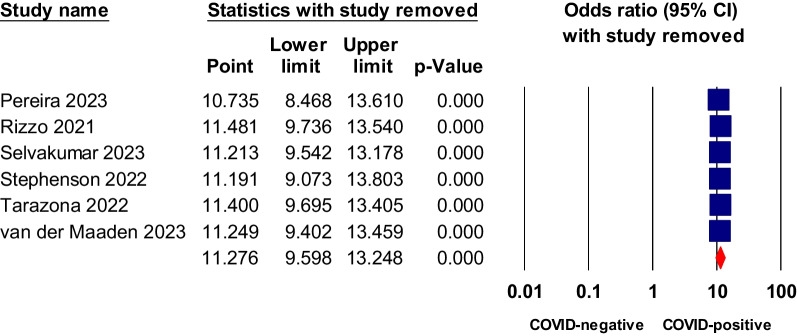
Sensitivity analysis of odds ratios of anosmia in non-hospitalized COVID-19 relative to negative control

**Fig. 8:**
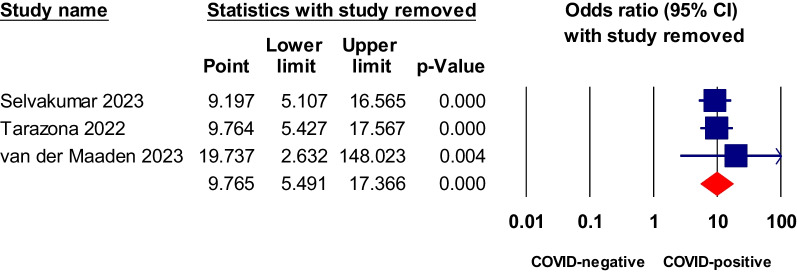
Sensitivity analysis of odds ratio of ageusia in non-hospitalized COVID-19 relative to negative control

**Fig. 9:**
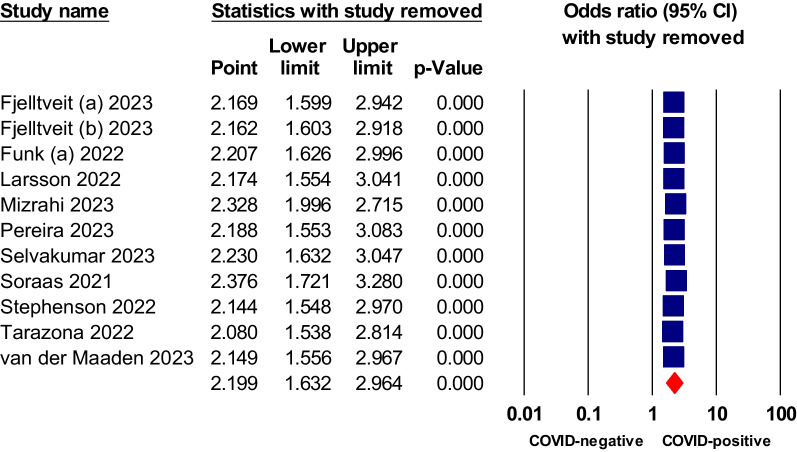
Sensitivity analysis of odds ratios of dyspnea in non-hospitalized COVID-19 relative to negative control

**Fig. 10:**
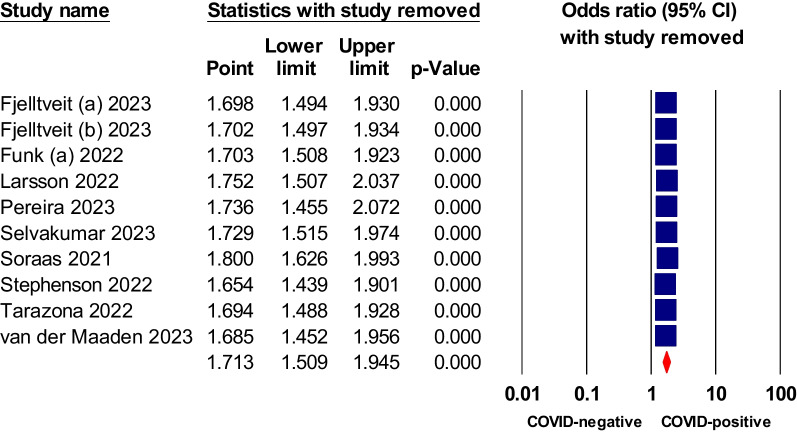
Sensitivity analysis of odds ratios of fatigue in non-hospitalized COVID-19 relative to negative control

**Fig. 11:**
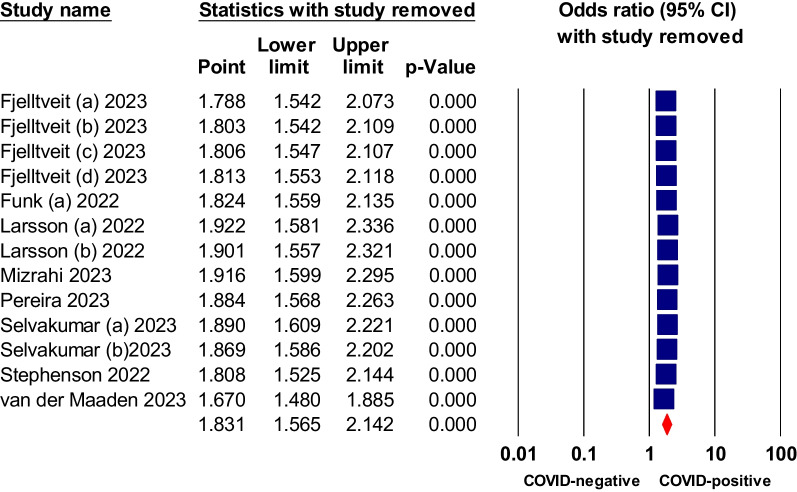
Sensitivity analysis of odds ratios of brain fog or memory deficits in non-hospitalized COVID-19 relative to negative control

We conducted a sensitivity analysis using the leave-one-out approach for symptoms or conditions that showed statistically significant higher odds in COVID patients relative to COVID-negative controls in both the matched comorbidity and overall analysis. Dyspnea, fatigue, brain fog, and anosmia all showed statistical significance in non-hospitalized COVID-19 relative to negative control. The analysis indicated that the pooled odds ratios of these tested symptoms were reliable and were not dependent on any study, as shown in Figs. 7, 8, 9, 10 and 11.

Publication bias testing was conducted for symptoms that displayed statistical significance and were present in at least 10 studies. The results of the funnel plots and Egger's tests did not provide any indications of publication bias for the three evaluated symptoms, namely dyspnea, fatigue, and brain fog, with corresponding p-values of 0.28, 0.58, and 0.09, respectively (Additional file 1: Figs. S1–S3).

## Discussion

The persistent symptoms after COVID-19 are non-specific and frequently reported among the general population. In addition, these symptoms may result from hospitalization for causes unrelated to COVID-19. In light of these considerations, we aimed to compare the burden of persistent symptoms after COVID-19 among individuals who tested positive and those who tested negative for SARS-CoV-2.

Our study highlighted the following main findings: (1) Analysis of symptoms in non-hospitalized COVID-19 patients compared to negative controls revealed that the majority of the tested symptoms were unrelated to COVID-19, and appeared to be equally prevalent in both groups. However, some symptoms, such as anosmia, ageusia, dyspnea, fatigue, and brain fog, seemed to be associated with COVID-19 in non-hospitalized patients. Particularly, anosmia and ageusia exhibited high odds ratios of 11.27 and 9.76, respectively. (2) The matched comorbidity subgroup analysis revealed that the presence of pre-existing medical conditions may influence the odds of experiencing specific symptoms and conditions in both children and adults. This was more evident in children than adults. However, it is important to note that the limited number of studies included in the analysis warrants caution in interpreting the findings. (3) The limited evidence so far indicates that there is no significant difference in cardiovascular abnormalities between individuals who had mild COVID-19 relative to seronegative healthy participants. But larger studies are still warranted to draw firmer conclusions. (4) Analysis of hospitalized COVID-19 patients compared to hospitalized patients for other indications did not show significantly higher odds of tested symptoms. Unexpectedly, headache and sleep disorders had higher odds of occurring in non-COVID hospitalized patients.

Similarly, a meta-analysis comparing the odds of post-COVID-19 symptoms in children relative to negative controls found that out of the 13 tested symptoms, persistent dyspnea, loss of smell or taste (anosmia/ageusia), and fever had odds ratios of 2.69, 10.68, and 2.23 respectively, indicating that these symptoms were significantly associated with COVID-19 infection [37]. However, the findings of this study may be limited by comparing COVID-infected children of varying severity to negative controls and the smaller number of the included studies (3 studies).

According to our analysis of symptoms in non-hospitalized COVID-19 patients relative to negative controls, anosmia exhibited a significantly high odds ratio of 11.27 in non-hospitalized COVID-19 patients when compared to the negative control group, which comprised individuals without any infection. Although anosmia was prevalent as a persistent symptom after COVID, it is challenging to determine whether it occurred more frequently than other upper respiratory tract infections (URTI) of similar severity. This is supported by the high frequency of post-viral olfactory disorders, which range from 11 to 40% [38]. Also, the prevalence of anosmia in the mixed hospitalized and non-hospitalized population of COVID survivors is 14.12% [13]. Furthermore, our meta-analysis of proportions showed that the prevalence of anosmia in both hospitalized and non-hospitalized COVID survivors is 14.3%. In addition, a recent study found that patients recovering from COVID-19 infections exhibited a lower incidence of complete anosmia compared to those recovering from non-COVID-19 infections [39]. Therefore, the uniqueness of anosmia to SARS‑CoV‑2 may be questioned.

Similarly, non-hospitalized COVID-19 patients were more likely to experience ageusia than negative controls, which also comprised individuals without any infection, with a pooled odds ratio of 6.57. According to a recent study, patients recovering from COVID-19 infections displayed ageusia less frequently than those recovering from non-COVID-19 infections [39]. Anosmia and ageusia are frequently reported after recovering from COVID-19; however, further research is necessary to determine conclusively if these chemosensory symptoms occur more often compared to other post-viral illnesses.

Non-hospitalized COVID-19 patients were also more likely to experience brain fog than negative controls in both adults and children. It is not yet clear whether to directly blame SARS-CoV2 itself for these symptoms or whether they are a result of particular idiopathic stressors. Moreover, there is no quantifiable standardized definition of brain fog, and the methodologies and outcome measures differ [40].

Fatigue is a frequently reported symptom of COVID-19 [41] and is considered the most frequent symptom among COVID survivors [13]. It is not unexpected that some individuals may experience fatigue after COVID-19 recovery, as post-infectious fatigue is a widely recognized phenomenon that has been observed in both viral and non-viral infections and is well-documented in the literature [42]. Conferring to our study, non-hospitalized COVID-19 patients have significantly higher odds of fatigue than negative controls, which persisted even after matching for comorbidity (with pooled odds of 2.2). However, it is important to note that fatigue is not limited to SARS-CoV-2 and has been observed in other infectious diseases, as mentioned earlier.

Similarly, dyspnea is strongly associated with non-hospitalized COVID-19 patients, even after matching for comorbidities. Although the specific mechanism responsible for dyspnea in mild COVID-19 survivors has yet to be determined, studies suggest that it is typically caused by hyperventilation rather than organ damage [43–47]. This hyperventilation may arise from an abnormality in ventilatory control or a failure of inhibitory systems (such as endorphins) following pulmonary infections [47].

From another perspective, persistent symptoms after COVID-19 may result from hospitalization for causes unrelated to COVID-19, which is referred to as post-hospital syndrome (PHS) or Post-intensive care syndrome (PICS). Throughout hospital stays, inpatients are subjected to considerable levels of stress, which might raise their risk for a wide range of adverse health events referred to as PHS [48]. Allostatic overload is thought to be the cause of PHS [48]. Additionally, the PICS has been identified as a distinct condition that specifically arises from ICU stays. PICS refers to a collection of physical, cognitive, and emotional symptoms that can occur in individuals who have survived a critical illness and received treatment in the ICU [49]. Either PHS or PICS could potentially explain why COVID-19 patients who have been hospitalized, do not exhibit a notably higher odds of tested symptoms compared to patients hospitalized for other causes. Interestingly, headaches and sleep disorders (OR 0.86 and 0.89, respectively, *P* < 0.05) showed significantly lower odds of occurrence in hospitalized COVID-19 patients compared to patients hospitalized for other reasons. However, the upper limits of the 95% CI nearly reach the null effect (one), as shown in Table 5. This borderline significance likely stems from the limited number of studies included (only three). With more studies, we could reach more definitive conclusions regarding this association. Similarly, Quinn et al. showed that the burden of post-acute physical and mental health disorders among patients who survived hospitalization for COVID-19 was comparable to that of other acute infectious diseases, suggesting that rather than being direct effects of the SARS-CoV-2 infection, many of the post-acute consequences of COVID-19 may be attributable to the severity of the infection illness requiring hospitalization [50]. Another study revealed that neurological risk was high in COVID-19 survivors, but not more than that observed after other infections of similar severity [51]. This highlights the importance of including well-matched control groups when investigating PCC.

## Limitations

This study has several limitations that should be considered when interpreting the results. These include the potential for heterogeneity due to differences in study design, population characteristics, and outcome definitions, as well as the risk of recall bias. Furthermore, some symptoms with borderline odds ratios warrant further investigation due to their limited research and occasionally smaller sample sizes, hindering their definitive association with PCC.

Future prospective studies aimed at achieving a more precise understanding and drawing explicit solid conclusions about the post-acute consequences of COVID-19 should consider several key factors. First, using a well-standardized clinical definition of outcomes instead of relying on patients’ self-reporting will increase the accuracy and reliability of study findings. Second, ensuring an appropriate sample size will increase the study's statistical power and improve the generalizability of the results. Third, matched control groups based on age, sex, and comorbidities should be included to account for potential confounding factors. Fourth, to ascertain COVID exposure, both serology and PCR to detect past and current COVID-19 infections can be used, respectively. The serology of the negative control should be repeated at least a month after the outcome assessment to check if seroconversion occurred. Fifth, considering historical and contemporary control to determine whether non-specific stressors related to the pandemic (such as social isolation, economic stress, and uncertainty) have any effect on PCC.

## Conclusion

In conclusion, the symptoms of PCC are non-specific and can be commonly reported among the general population and post-upper respiratory tract infections. In addition, many of these symptoms may also result from hospitalization for causes unrelated to COVID-19. Therefore, the exclusivity of PCC as a consequence of the SARS-CoV-2 infection is questioned.

### Supplementary Information

**Additional file 1**. **Table S1**: Supplementary preferred reporting items for systematic reviews and meta-analyses (PRISMA) checklist; **Table S2**: Full search strategy; **Table S3**: Checklist items for quality assessment of the included studies; **Table S4**: List of excluded studies; **Table S5**: Quality assessment of the included studies; **Table S6**: Pooled odds ratios for clinical signs and symptoms across all included studies stratified by patients’ age category regardless the hospitalization state; **Fig. S1**: Funnel plot of dyspnea in non-hospitalized COVID-19 patients relative to negative control; **Fig. S2**: Funnel plot of fatigue in non-hospitalized COVID-19 patients relative to negative control; **Fig. S3**: Funnel plot of brain and memory deficits in non-hospitalized COVID-19 patients relative to negative control.

## Data Availability

All data generated and or analyzed throughout this study were included either in this published article or its supplementary information file.

## Abbreviations

CI: Confidence interval
COPD: Chronic obstructive pulmonary disease
COVID: Coronavirus disease
COVID-19: Coronavirus disease 2019
CT: Computed tomography
ICU: Intensive care unit
OR: Odds ratio
PC: Prospective cohort
PCC: Post-COVID condition
PCR: Polymerase chain reaction
PHS: Post-hospital syndrome
PICS: Post-intensive care syndrome
PRISMA: Preferred reporting items for systematic reviewers and meta-analysis
RC: Retrospective cohort
SARS-CoV-2: Severe acute respiratory syndrome coronavirus 2
URTI: Upper respiratory tract infection
WHO: World health organization
