# Supplementary material for: The burden of persistent symptoms after COVID-19 (long COVID): a meta-analysis of controlled studies in children and adults
Source: Virol J. 2024 Jan 11;21:16. doi: 10.1186/s12985-024-02284-3 (PMC10785462; doi:10.1186/s12985-024-02284-3)
Supplement: Supplementary file 1 — Additional file 1. Table S1: Supplementary preferred reporting items for systematic reviews and meta-analyses (PRISMA) checklist; Table S2: Full search strategy; Table S3: Checklist items for quality assessment of the included studies; Table S4: List of excluded studies; Table S5: Quality assessment of the included studies; Table S6: Pooled odds ratios for clinical signs and symptoms across all included studies stratified by patients’ age category regardless the hospitalization state; Fig. S1: Funnel plot of dyspnea in non-hospitalized COVID-19 patients relative to negative control; Fig. S2: Funnel plot of fatigue in non-hospitalized COVID-19 patients relative to negative control; Fig. S3: Funnel plot of brain and memory deficits in non-hospitalized COVID-19 patients relative to negative control. [file 12985_2024_2284_MOESM1_ESM.docx]

**The burden of persistent symptoms after COVID-19 (long COVID): A Meta-analysis of controlled studies in children and adults**

1Department of Ahmed Azzam^1*^, Heba Khaled^2^, Neveen Refaey^3^, Shorouk Mohsen^4^, Ola Ali El-Emam^5^, Nada Dawood^6^, Hebatalla A. Ahmed^7^, Omar A. Soliman^8,9^, Sana Mostafa^10^, Heba Ramadan^11^, Maha Mosa^12^, Amora Omar Ibrahim Elmowafy^13^, Shimaa Mohamed Abdou Rizk^13^, Ahmed Zaki^14^, Mostafa Hussien^15^, Ameer Ahmed^16^, Ahmad Ashraf Ezzat^17^, Fatma E. Hassan^18,19^

^1^Department of Microbiology and Immunology, Faculty of Pharmacy, Helwan University, Cairo, Egypt.
^2^Department of Biochemistry, Faculty of Pharmacy, Cairo University, Cairo, Egypt.
^3^Department of Physical Therapy for Women's Health, Faculty of Physical Therapy, Cairo University, Cairo, Egypt.
^4^Public Health and Preventive Medicine Department, Faculty of Medicine, Mansoura University, Mansoura, Egypt.
^5^Clinical Pathology Department, Faculty of Medicine, Mansoura University, Mansoura, Egypt.
^6^Community Medicine Department, Faculty of Medicine, Suez Canal University, Ismailia, Egypt.
^7^Department of Public Health and Community Medicine, Faculty of Medicine, Kafr-Elsheikh University, Kafr-Elsheikh, Egypt.
^8^Department of Clinical Pharmacy, Alexandria, University Main Teaching Hospital, Alexandria, Egypt.
^9^Human Genetics Department, Medical Research Institute, Alexandria University, Alexandria, Egypt.
^10^Oral Biology Department, Faculty of Dentistry, Cairo University, Cairo, Egypt.
^11^Pharmacy Department, Agamy Medical District, Ministry of Health and Population, Agamy, Alexandria, Egypt.
^12^Otolaryngologist, Qeft Teaching Hospital, Qena, Egypt.
^13^Medical Surgical Nursing Department, Faculty of Nursing, Mansoura University, Mansoura, Egypt.
^14^Faculty of Medicine, Cairo University, Cairo, Egypt.
^15^Faculty of Medicine, Suez Canal University, Suez City, Ismailia governorate, Egypt.
^16^Faculty of Medicine, Minia University, Minya, Egypt.
^17^Faculty of Pharmacy, Minia University, Minya, Egypt ^18^Medical Physiology Department, Kasr Alainy, Faculty of Medicine, Cairo University, Giza 11562, Egypt.
^19^ Department of Physiology, General Medicine Practice Program, Batterjee Medical College, Jeddah 21442, Saudi Arabia.

***Corresponding author: Ahmed Azzam**

Department of Microbiology and Immunology, Faculty of Pharmacy, Helwan University, Cairo Egypt.

Telephone: ‎+20 0237222210

Email: ahmed.abdelkareem@pharm.helwan.edu.eg

**Telephone:** ‎+20 0237222210

**Email:** [ahmed.abdelkareem@pharm.helwan.edu.eg](mailto:ahmed.abdelkareem@pharm.helwan.edu.eg)


**Contents**

| **Table No.** | **Table title** | **Page** |
| --- | --- | --- |
| **Table S1** | Supplementary preferred reporting items for systematic reviews and meta-analyses (PRISMA) checklist | 3-5 |
| **Table S2** | Full search strategy | 6 |
| **Table S3** | Checklist items for quality assessment of the included studies | 6,7 |
| **Table S4** | List of excluded studies | 7-11 |
| **Table S5** | Quality assessment of the included studies | 11,12 |
| **Table S6** | Pooled odds ratios for clinical signs and symptoms across all included studies stratified by patients’ age category | 13 |
| **Figs. S1-3** | Publication bias testing | 14-16 |

**Table S1:** Supplementary preferred reporting items for systematic reviews and meta-analyses (PRISMA) checklist.

| Section/topic | Item No | Checklist item | Reported on page No. |
| --- | --- | --- | --- |
| Title | | | |
| Title | 1 | Identify the report as a systematic review, meta-analysis, or both | 1 |
| Abstract | | | |
| Structured summary | 2 | Provide a structured summary including, if applicable, background, objectives, data sources, study eligibility criteria, participants, interventions, study appraisal and synthesis methods, results, limitations, conclusions, implications of key findings, and systematic review registration number | 2,3 |
| Introduction | | | |
| Rationale | 3 | Describe the rationale for the review in the context of what is already known | 4,5 |
| Objectives | 4 | Provide an explicit statement of questions being addressed concerning participants, interventions, comparisons, outcomes, and study design (PICOS) | 5,6 |
| Methods | | | |
| Protocol and registration | 5 | Indicate if a review protocol exists, if and where it can be accessed (such as web address), and, if available, provide registration information including registration number | - |
| Information sources | 6 | Describe all information sources (such as databases with dates of coverage and contact with study authors to identify additional studies) in the search and date last searched | 6 |
| Eligibility criteria | 7 | Specify study characteristics (such as PICOS and length of follow-up) and report characteristics (such as years considered, language, and publication status) used as criteria for eligibility and give a rationale | 6,7 |
| Search | 8 | Present a full electronic search strategy for at least one database, including any limits used, such that it could be repeated | Table S2 |
| Study selection | 9 | State the process for studies selection in regards to screening, eligibility, inclusion in the systematic review, and, if applicable, inclusion in the meta-analysis | 6,7 |
| Data collection process | 10 | Describe the method of data extraction from reports (such as piloted forms, independently, in duplicate) and any processes for obtaining and confirming data from investigators | 8 |
| Data items | 11 | List and define all variables for which data were sought (such as PICOS and funding sources) and any assumptions and simplifications made | 8 |
| Risk of bias in individual studies | 12 | Describe methods used for assessing the risk of bias in individual studies (including specification of whether this is done at the study or outcome level), and how this information is to be used in any data synthesis | 8 |
| Summary measures | 13 | State the principal summary measures (such as risk ratio and difference in means) | 8 |
| Synthesis of results | 14 | Describe the methods of handling data and combining results of studies, if done, including measures of consistency (such as I^2^ statistic) for each meta-analysis | 9 |
| Risk of bias across studies | 15 | Specify any assessment of the risk of bias that may affect the cumulative evidence (such as publication bias and selective reporting within studies) | 9 |
| Additional analyses | 16 | Describe methods of additional analyses (such as sensitivity or subgroup analyses and meta-regression), if done, indicating which were pre-specified | 9 |
| Results | | | |
| Study selection | 17 | Give numbers of studies screened, assessed for eligibility, and included in the review, with reasons for exclusions at each stage, ideally with a flow diagram | 10 |
| Study characteristics | 18 | For each study, present characteristics for which data were extracted (such as study size, PICOS, and follow-up period) and provide the citations | 10  Table 1 |
| Risk of bias within studies | 19 | Present data on the risk of bias of each study and, if available, any outcome-level assessment (See item 12) | Tables S3 and S4 |
| Results of individual studies | 20 | For all outcomes considered (benefits or harms), present for each study (a) Simple summary data for each intervention group and (b) Effect estimates and confidence intervals, ideally with a forest plot | 13-17  Tables 2-6 Figs. 2-6 |
| Synthesis of results | 21 | Present results of each meta-analysis done, including confidence intervals and measures of consistency | 13-17  Table 2-6 Figs. 2-6 |
| Risk of bias across studies | 22 | Present results of any assessment of the risk of bias across studies (See item 15) | 18  Figs. S1-S3 |
| Additional analysis | 23 | Give results of additional analyses, if done (such as sensitivity or subgroup analyses and meta-regression) (See item 16) | 18  Figs. 7-11 |
| Discussion | | | |
| Summary of evidence | 24 | Summarize the main findings including the strength of evidence for each main outcome; consider their relevance to key groups (such as health care providers, users, and policymakers) | 18-22 |
| Limitations | 25 | Discuss limitations at the study and outcome level (such as the risk of bias), and review level (such as incomplete retrieval of identified research and reporting bias) | 22,23 |
| Conclusions | 26 | Provide a general interpretation of the results in the context of other evidence, and implications for future research | 23 |
| Funding | | | |
| Funding | 27 | Describe sources of funding for the systematic review and other support (such as the supply of data) and the role of funders for the systematic review | 25 |

**Table S2:** Full search strategy.

| **Search string** | |
| --- | --- |
| **#1** | "Post-acute sequelae of SARS-CoV-2 infection" OR "PASC" OR "Post-acute sequelae of COVID-19" OR "Long COVID" OR "Long Haul COVID" OR "Long-Haul COVID" OR "COVID long Haulers" OR "Chronic COVID syndrome" OR "Post-COVID syndrome" OR "Post-acute COVID-19 syndrome" OR "Post-acute COVID syndrome" OR “PACS” OR “Post-Covid condition” OR “PCC” |
| **#2** | “COVID negative control” OR “Non-COVID control” OR “Healthy control” OR “Non-exposed control” OR “Unexposed control" OR “Non-infected control” OR “Negative-tested control” |
| **#3** | #1 and #2 |

**Table S3:** Checklist items for quality assessment of the included studies.

| **Checklist Item** | **Response** |
| --- | --- |
| **1. Sample size^β^** | |
| a) Exceeds 10,000 | Yes* |
| b) Exceeds 10,000 | No |
| **2. Ascertainment of exposure of the case** | |
| a) PCR test | Yes* |
| b) Serology test | Yes* |
| c) Both | ** |
| d) None |  |
| **3. Same method of ascertainment for negative control** | |
| a) Yes | Yes* |
| b) No |  |
| **4. Definition of controls^α^** | |
| a) No history of the disease (endpoint) | Yes* |
| b) No description of the source | |
| **5. Non-response rate** | |
| a) Same rate for both groups | Yes* |
| b) Non-respondents described | |
| c) Rate different and no designation | |
| **6. Assessment of symptoms** | |
| a) Self-reported | |
| b) Scale-based or Physician assessment | ** |
| **7. Control for comorbidity, age, and gender** | |
| a) Yes | Yes** |
| b) No |  |
| **8. If (7) is “No”, does the study adjust for confounding factors or use stratified sampling?** | |
| a) Yes | Yes ** |
| b) No |  |

**Notes:**

**β**: There was a nearly similar proportion of post-COVID symptoms in COVID-positive individuals relative to COVID-negative individuals, which mandates a higher sample size to allow accurate estimation of odds ratios. Therefore, a limit of 10,000 was set to ensure adequate statistical power. *: Denotes 1 mark.

**: Denotes 2 marks.

**α:** This item did not apply to studies that compared hospitalized COVID patients to hospitalized non-COVID patients.

**Table S4: List of excluded studies**

| **DOI** | **Author** | **Reasons for exclusion** |
| --- | --- | --- |
| <https://doi.org/10.3238%2Farztebl.m2022.0134> | Schulz | The study cases were mixed (hospitalized and non-hospitalized) compared to the negative control, and no stratification based on hospitalization was performed. |
| <https://doi.org/10.1001/jamapediatrics.2022.2800> | Rao | The study cases were mixed (hospitalized and non-hospitalized) compared to the negative control, and no stratification based on hospitalization was performed. |
| <https://doi.org/10.3201%2Feid2905.221349> | Asakura | The study cases were mixed (hospitalized and non-hospitalized) compared to the negative control, and no stratification based on hospitalization was performed. |
| <https://doi.org/10.1016/S0140-6736(22)01214-4> | Ballering | The study cases were mixed (hospitalized and non-hospitalized) compared to the negative control, and no stratification based on hospitalization was performed (2.7% of hospitalized COVID cases compared with healthy controls). |
| <https://doi.org/10.1016/S2352-4642(22)00004-9> | Berg | Outcomes assessment is less than 3 months since infection |
| <https://doi.org/10.1016/j.gastha.2023.06.006> | Blackett | The study cases were mixed (hospitalized and non-hospitalized) compared to the negative control, and no stratification based on hospitalization was performed.  Among those with COVID-19, 15% were hospitalized, and 1.7% required mechanical ventilation. None of the COVID-19-negative patients were hospitalized.  No outcome of non-hospitalized cases compared with healthy controls was reported. |
| <https://doi.org/10.1007/s00431-021-04345-z> | Borch | The study cases were mixed (hospitalized and non-hospitalized) compared to the negative control, and no stratification based on hospitalization was performed. |
| <https://doi.org/10.1016/j.eclinm.2021.101159> | Cassar | The study cases were hospitalized, and the control group was non-hospitalized (100% hospitalized cases compared to non-hospitalized controls). |
| <https://doi.org/10.7189%2Fjogh.13.06015> | Cazé | Outcomes assessment is less than 3 months since infection |
| <https://doi.org/10.1093/cid/ciab338> | Chevinsky | Outcomes assessment is less than 3 months since infection |
| <https://doi.org/10.1136/bmjopen-2022-064979> | Donnachie | The study cases were mixed (hospitalized and non-hospitalized) compared to the negative control, and no stratification based on hospitalization was performed (unclear hospitalization status of cases). |
| <https://doi.org/10.1038/s41467-022-34616-8> | Dumont | The study did not clarify the hospitalization status of the cases. |
| <https://doi.org/10.2337/db21-0329> | Fernández-de-las-Peñas | The focus was on diabetic patients. |
| <https://doi.org/10.1111%2Fjgh.15717> | Ghoshal | The study cases were mixed (hospitalized and non-hospitalized) compared to the negative control, and no stratification based on hospitalization was performed. |
| <https://doi.org/10.1016/j.cgh.2022.10.015> | Golla | The study cases were mixed (hospitalized and non-hospitalized) compared to the negative control, and no stratification based on hospitalization was performed (3.4% severe and 22.1% moderate cases compared with healthy controls). |
| <https://doi.org/10.1038/s41467-022-33415-5> | Hastie | The study cases were mixed (hospitalized and non-hospitalized) compared to the negative control, and no stratification based on hospitalization was performed. |
| <https://doi.org/10.1016/j.ajp.2022.103409> | HE | The study cases were hospitalized compared to the negative control, and no stratification based on hospitalization was performed. |
| <https://doi.org/10.1016/S0140-6736(21)01755-4> | Huang | The study cases were hospitalized compared to the negative control, and no stratification based on hospitalization was performed. |
| <https://doi.org/10.3390/pathogens10101246> | Kozak | There was no control group. |
| <https://doi.org/10.3389/fpubh.2022.975992> | Líška | The study did not clarify the hospitalization status of the cases. |
| <https://doi.org/10.1111/echo.15199> | Mahajan | Outcomes assessment is less than 3 months since infection |
| <https://doi.org/10.14309/ajg.0000000000001541> | Marasco | Outcomes assessment is less than 3 months since infection |
| <https://doi.org/10.1007/s00415-021-10579-6> | Mattioli | The study cases were hospitalized compared to the negative control, and no stratification based on hospitalization was performed (2% of COVID patients were hospitalized). |
| <https://doi.org/10.1016/j.dsx.2021.102302> | Mittal | The focus was on diabetic patients. |
| <https://doi.org/10.1016/S2352-4642(21)00198-X> | Molteni | Outcomes assessment is less than 3 months since infection |
| <https://doi.org/10.3390/healthcare9050575> | Orru | The study did not clarify the hospitalization status of the cases. |
| <https://doi.org/10.30773/pi.2021.0223> | Park | The study cases were mixed (hospitalized and non-hospitalized) compared to the negative control, and no stratification based on hospitalization. |
| <https://doi.org/10.1007/s00405-021-06764-y> | Riestra | The study does not clarify the hospitalization status of the cases. |
| <https://doi.org/10.1371/journal.pmed.1004122> | Roessler | The study cases were mixed (hospitalized and non-hospitalized) compared to the negative control, and no stratification based on hospitalization was performed (cases: adults: 5.8% hospitalized and 2.8% ICU; children: 1% hospitalized and 0.4% ICU - i.e., total more than 1%). |
| <https://doi.org/10.1136/heartjnl-2021-319926> | Singh | The study cases were hospitalized compared to the negative control, and no stratification based on hospitalization was performed. |
| <https://doi.org/10.1038/s41467-022-31897-x> | Sørensen | The study cases were hospitalized compared to the negative control, and no stratification based on hospitalization was performed. |
| <https://doi.org/10.1371/journal.pone.0278057> | Tisler | The study cases were hospitalized compared to the negative control, and no stratification based on hospitalization was performed. |
| <https://doi.org/10.1016/j.amjmed.2023.04.022> | Xuereb | The study cases were hospitalized compared to the negative control, and no stratification based on hospitalization was performed (9.2% of COVID cases were hospitalized). |
| <https://doi.org/10.1093%2Fcid%2Fciab991> | Zavala | The study did not clarify the hospitalization status of the cases, and Outcomes assessment is less than 3 months since infection. |
| <https://doi.org/10.1186/s12916-021-02115-0> | Estiri | Outcomes assessment is less than 3 months since infection |
| <https://doi.org/10.1186/s12889-023-16026-7> | Cox | Outcomes assessment is less than 3 months since infection |
| <https://doi.org/10.1136/bmj.p932> | Ballouz | The study cases were mixed (hospitalized and non-hospitalized) compared to the negative control, and no stratification based on hospitalization was performed. |
| <https://doi.org/10.1093/cid/ciac947> | Nehme | The study cases were mixed (hospitalized and non-hospitalized) compared to the negative control, and no stratification based on hospitalization was performed |
| <https://doi.org/10.15585%2Fmmwr.mm7131a3> | Kompaniyets | Outcomes assessment is less than 3 months since infection |
| <https://doi.org/10.1016/S2215-0366(21)00084-5> | Taquet | Outcomes assessment is less than 3 months since infection |
| <https://doi.org/10.1038/s41591-022-01689-3> | Xie | Outcomes assessment is less than 3 months since infection |
| doi:10.1001/jamainternmed.2023.2228 | Quinn | Outcomes assessment is less than 3 months since infection |
| <https://doi.org/10.1038/s41467-023-36223-7> | XU | Outcomes assessment is less than 3 months since infection |

**Table S5:** Quality assessment of the included studies.

| **Author** | **Checklist item^α^** | | | | | | | | | | | | | | | | | | | **Score**  **(Out of 10)** |
| --- | --- | --- | --- | --- | --- | --- | --- | --- | --- | --- | --- | --- | --- | --- | --- | --- | --- | --- | --- | --- |
|  | **1** | | **2** | | | | **3** | | **4** | | **5** | | | **6** | | **7** | | **8** | |  |
|  | **a** | **b** | **a** | **b** | **c** | **d** | **a** | **b** | **A** | **b** | **a** | **b** | **c** | **a** | **b** | **a** | **b** | **a** | **b** |  |
| Larsson | **1** |  |  | 1 |  |  | **1** |  | 1 |  | **1** |  |  |  | 2 | 2 |  |  |  | 9 |
| Pereira | **1** |  | 1 |  |  |  | **1** |  | 1 |  | **1** |  |  |  | 2 | 2 |  |  |  | 9 |
| Selvakumar | **1** |  |  |  | 2 |  | **1** |  | 1 |  |  |  |  |  | 2 |  |  | 2 |  | 9 |
| Fjelltveit |  | **0** |  |  | 2 |  | **1** |  | 1 |  | **1** |  |  |  | 2 | 2 |  |  |  | 9 |
| Gorecka |  | **0** |  |  | 2 |  | **1** |  | 1 |  |  | **0** |  |  | 2 | 2 |  |  |  | 8 |
| Joy |  | **0** |  |  | 2 |  | **1** |  | 1 |  |  | **0** |  |  | 2 | 2 |  |  |  | 8 |
| Mizrahi | **1** |  | 1 |  |  |  | **1** |  | 1 |  |  | **0** |  |  | 2 |  |  | 2 |  | 8 |
| van der Maaden | **1** |  |  |  | 2 |  | **1** | **0** | 1 |  |  |  | **1** | 0 |  | 2 |  |  |  | 8 |
| Castro | **1** |  | 1 |  |  |  | **1** |  | NA | NA |  | **0** |  |  | 2 | 2 |  |  |  | 7 |
| Funk |  | **0** | 1 |  |  |  | **1** |  | 1 |  | **1** |  |  |  |  |  |  | 2 |  | 6 |
| Liptaka |  | **0** | 1 |  |  |  | **1** |  | 1 |  | **1** |  |  | 0 |  | 2 |  |  |  | 6 |
| Soraas |  | **0** | 1 |  |  |  | **1** |  | 1 |  |  |  | **0** | 0 |  |  |  | 2 |  | 6 |
| Stephenson | **1** |  | 1 |  |  |  | **1** |  | 1 |  |  |  |  |  |  | 2 |  |  |  | 6 |
| Tarazona |  | **0** | 1 |  |  |  | **1** |  | 1 |  |  |  | **0** |  | 2 |  |  |  |  | 5 |
| Boscolo-Rizzo |  | **0** | 1 |  |  |  | **1** |  | 1 |  |  | **0** |  |  | 2 |  |  |  |  | 5 |
| Nersesjan |  | **0** | 1 |  |  |  |  |  | NA | NA |  |  | **0** |  | 2 | 2 |  |  |  | 5 |
| Rivera‑Izquierdo |  | **0** | 1 |  |  |  | **1** |  | NA | NA | **1** |  |  | 0 |  | 2 |  |  |  | 5 |
| Radtke | **0** |  |  | 1 |  |  | **1** |  | 1 |  |  | **0** |  | 0 |  | 2 |  |  |  | 5 |
| Elkan |  | **0** | 1 |  |  |  | **1** |  | NA | NA |  | **0** |  | 0 |  | 2 |  |  |  | 4 |
| Blankenburg |  | **0** |  | 1 |  |  | **1** |  | 1 |  |  | **0** |  | 0 |  |  | 0 |  | 0 | 3 |

**Notes:**
α: Indicates that the complete details of checklist items are presented in Table S3
NA: Non-applicable checklist item as the study aimed to evaluate post-COVID condition (PCC) in hospitalized COVID-19 patients compared to those hospitalized due to other indications.

**Table S6:** Pooled odds ratios for clinical signs and symptoms across all included studies stratified by patients’ age category regardless the hospitalization state.

|  | **Overall** | | | **Adults** | | | **Children** | | |
| --- | --- | --- | --- | --- | --- | --- | --- | --- | --- |
| **Clinical signs/symptoms** | **Studies**  **(No.)** | **Odds ratio  (95% CI)** | **I^2^%** | **Studies**  **(No.)** | **Odds Ratio  (95% CI)** | **I^2^%** | **Studies**  **(No.)** | **Odds ratio  (95% CI)** | **I^2^%** |
| Anosmia | 8 | 6.9 (2.54-18.6) | 97.6 | 5 | 4.37 (1.23-15.49) | 95.5 | 2 | 11.59 (9.71-13.85) | 0 |
| Dyspnea | 13 | 2.1 (1.59-2.78) | 95.5 | 9 | 1.88 (1.46-2.42) | 89.5 | 6 | 1.6 (1-2.5) | 97.5 |
| Chest pain | 10 | 1.72 (1.18-2.51) | 92.9 | 6 | 1.36 (0.98-1.9) | 94 | 6 | 1.33 (0.85-2.1) | 94.1 |
| Brain fog/Confusion/Difficulty in concentration | 21 | 1.77 (1.48-2.12) | 95.2 | 12 | 2.04 (1.6-2.61) | 93.2 | 6 | 1.65 (1.28-2.12) | 94.3 |
| Dizziness | 7 | 1.35 (1.06-1.72) | 92 | 3 | 1.18 (1.03-1.34) | 66.8 | 6 | 1.38 (1.05-1.82) | 86.2 |
| Tachycardia /Palpitation | 4 | 1.34 (1.04-1.73) | 88.07 | 4 | 1.34 (1.04-1.73) | 66.2 | - | - |  |
| Ageusia | 4 | 6.57 (2.2-19.2) | 62.7 | 4 | 6.57 (2.2-19.2) | 62.7 | - | - |  |
| Fatigue | 15 | 1.53 (1.24-1.87) | 92.2 | 9 | 1.39 (1.07-1.82) | 60.1 | 5 | 1.85 (1.57-2.18) | 92.4 |
| Myalgia/Arthralgia | 14 | 1.22 (1.05-1.41) | 88.8 | 9 | 1.09 (0.96-1.22) | 82.6 | 8 | 1.17 (0.73-1.87) | 91 |
| Nausea/Vomiting | 5 | 0.95 (0.81-1.12) | 51.7 | 6* | 0.94 (0.83 -1.07 | 35.6 | - | - |  |
| Abdominal pain | 8 | 0.91 (0.71-1.17) | 86.9 | 6 | 0.79 (0.65-0.96) | 83.08 | 4 | 0.99 (0.74-1.33) | 93.1 |
| Sore throat | 9 | 0.85 (0.67-1.08) | 90.5 | 6 | 0.78 (0.56-1.08) | 91.8 | 5 | 1.22 (1.01-1.47) | 67.6 |
| Headache | 14 | 1.09 (0.89-1.33) | 94.3 | 8 | 0.9 (0.78-1.05) | 86.9 | 7 | 1.22 (0.88-1.69) | 95.7 |
| Congested or runny nose | 5 | 0.89 (0.78-1.02) | 0 | 3 | 0.9 (0.78-1.03) | 0 | 5 | 0.71 (0.42-1.2) | 0 |
| Heartburn/Stomachache | 3 | 1.02 (0.9-1.16) | 0 | - | - |  | - | - |  |
| Fever | 8 | 1.02 (0.82-1.27) | 32.5 | 3 | 0.99 (0.62-1.5) | 72.8 | 4 | 0.97 (0.77-1.22) | 0 |
| Anxiety | 11 | 0.94 (0.76-1.15) | 83.7 | 7 | 0.96 (0.75-1.24) | 89.6 | 4 | 0.82 (0.62-1.09) | 0 |
| Insomnia /Sleep disorder | 9 | 1.01 (0.88-1.15) | 54.3 | 7 | 1.04 (0.88-1.22) | 60.8 | - | - |  |
| Depression | 9 | 0.95 (0.78-1.16) | 50 | 6 | 1.08 (0.98-1.19) | 0 | 3 | 0.99 (0.26-3.73) | 0 |
| Diarrhea | 6 | 1.03 (0.78-1.37) | 73.5 | 4 | 0.76 (0.3-1.9) | 78.6 | - | - |  |
| Cough | 11 | 0.95 (0.81-1.12) | 78.7 | 6 | 0.9 (0.74-1.1) | 88.7 | 7 | 0.97 (0.88-1.07) | 18.7 |
| Ear problems/Earache/Ringing in ears | 6 | 1.23 (0.97-1.55) | 90.4 | 5 | 1.05 (0.83-1.32) | 86.1 | 4 | 0.96 (0.61-1.5) | 84.2 |

**NOTE: *** The number of estimates in the adult subgroup exceeded the overall, as Mizrahi et al. contributed one estimate to the overall analysis and two estimates to the adult subgroup analysis (19–40 years, 41–60 years).
**Abbreviations:** No.; Number, CI; Confidence interval

**Fig. S1: Funnel plot of dyspnea in non-hospitalized COVID-19 patients relative to negative control. The P value of Egger's tests is 0.28.**

**Fig. S2: Funnel plot of fatigue in non-hospitalized COVID-19 patients relative to negative control. The P value of Egger's tests is 0.58.**

**Fig. S3: Funnel plot of brain and memory deficits in non-hospitalized COVID-19 patients relative to negative control. The P value of Egger's tests is 0.09.**
